# Synthesis of hydroxyapatite from eggshells *via* wet chemical precipitation: a review

**DOI:** 10.1039/d4ra02198c

**Published:** 2024-07-08

**Authors:** Zaid Kareem, Ersan Eyiler

**Affiliations:** a Prosthetics and Orthotics Engineering Department, University of Kerbala Iraq zaltaey88@gmail.com; b Advanced Materials and Nanotechnology Department, Cukurova University Adana Turkey eeyiler@gmail.com; c Department of Chemical Engineering, Cukurova University Adana Turkey; d Tissue Engineering Department, Cukurova University Adana Turkey

## Abstract

In conjunction with the global trend towards sustainable industry, this review provides a summary of the research endeavors and efforts made in the field of exploiting eggshells in the production of hydroxyapatite (HA). HA is one of the most used biomaterials and has attracted considerable attention over the years towards biomedical applications. As the traditional production of HA from calcium and phosphorus chemical precursors synthetically has bottlenecks of being expensive, complex, time consuming, and results in a low biocompatible product, natural resources have become an attractive alternative option to synthesize HA, with trace elements providing a higher performance. Eggshell, with a growing production annually, is potentially a promising natural resource for HA production. Many studies have used different wet chemical precipitation routes to produce HA with properties comparable to synthetic HA. Thus, this review provides an overview of the various routes that can be used to synthesize HA from eggshells. In this review, the synthesis of HA from eggshells *via* wet chemical precipitation methods is specifically discussed in term of synthesis parameters and properties of the synthesized HA. This review should aid in choosing the most suitable route for HA production with the optimum parameters for obtaining the desired properties to meet the requirements of biomedical applications such as tissue engineering.

## Abstract 2

In conjunction with the global trend towards sustainable industry, this review provides a summary of the research endeavors and efforts made in the field of exploiting eggshells in the production of hydroxyapatite (HA).

## Graphical abstract

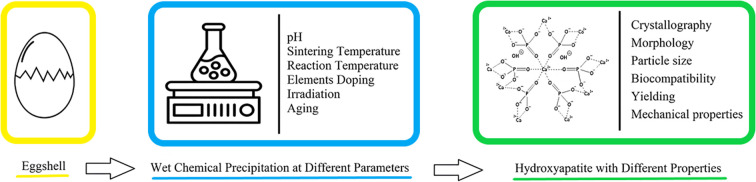

## 1. Introduction

**Fig. 1:**
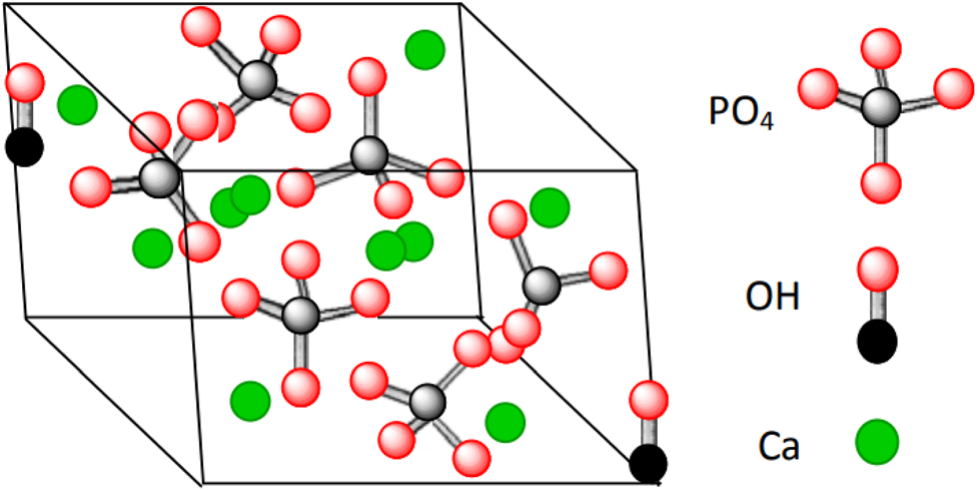
Crystal structure of hydroxyapatite showing the distribution of Ca : PO_4_ : OH with an emphasis on the Ca/P ratio of 1.6.^10^

Hydroxyapatite (HA) is a mineral form of calcium apatite that exhibits excellent biocompatibility and is a promising candidate for bone tissue repair and substitution.^1–4^ HA is a ceramic material composed of calcium phosphate, thus is nontoxic, and it is the main part of bone and teeth tissues.^2,5–7^ In 1926, Jong, W. derived the chemical formula of HA as Ca_10_(PO_4_)_6_(OH)_2_,^8,9^ constituting 39% by weight Ca, 18.5% P, and 3.38% OH (Fig. 1).^11^ Hydroxyapatite crystallizes in a hexagonal system, though with some exceptions in a monoclinic system.^12,13^ HA's structure is formed by a tetrahedral arrangement of phosphate ions (PO_4_^3−^), which constitute the “skeleton” of the unit cell, in which two of the four oxygens are aligned with the *c* axis and the other two are in a horizontal plane.^11^

**Fig. 2:**
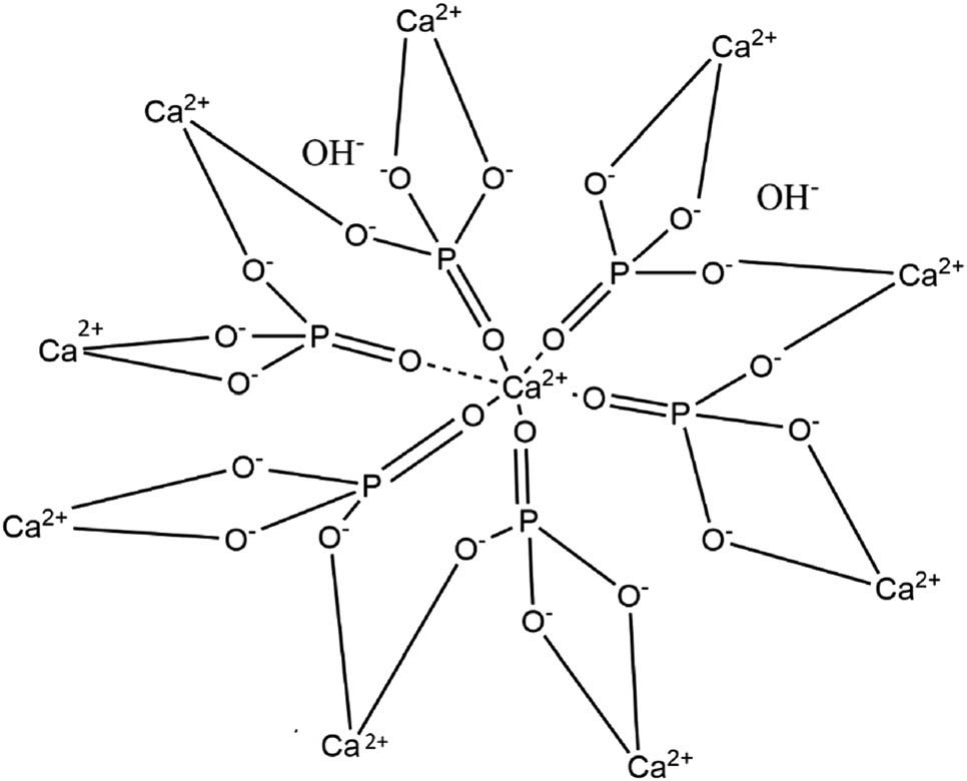
Molecular structure of hydroxyapatite. Apatites are chemical compounds of phosphorous either with hydroxyl (OH^−^), chloride (Cl^−^), or fluoride (F^−^) ions.^19^

Hydroxyapatite is the main inorganic component of bones (constituting about 70% of bone tissue).^14–17^ Stoichiometric HA is basically composed of calcium and phosphorus with the molar ratio of Ca/P equal to 1.67,^18^ and this ratio has been proven to be the most effective in promoting bone regeneration (Fig. 2).^20–22^ Natural HA has been shown to be deficient in calcium^23^ and phosphate.^24^ Synthetic HA is similar to biological apatite (or osseous mineral); however, there are some differences in its chemical composition and crystallinity.^25^ Compared to synthetic HA, natural HA is non-stoichiometric since it contains trace elements such as Na^+^, Zn^2+^, Mg^2+^, K^+^, Si^2+^, Ba^2+^, F^−^, and CO_3_^2−^, which make it similar to the chemical composition of human bone.^20^ Valuable trace ions in extracted HA play a crucial role in the bone regeneration process and are well known to accelerate the bone-formation process.^26,27^ Synthetic HA with one or more trace ions can be produced with a laborious process; however, ion-substituted HA's cost is markedly more expensive than simple HA.^20,28–30^

**Fig. 3:**
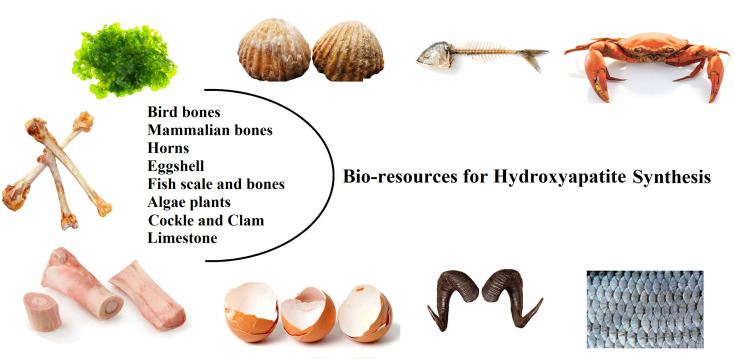
Natural resources for hydroxyapatite synthesis.

HA extracted from natural sources can be used to fabricate these materials with a sustainable, economical process that is also environmentally friendly because they are available as large quantity resources.^31–33^ This can result in positive contributions to the economy, environment, and general health. Natural hydroxyapatite is usually extracted from biological sources or wastes, such as shell sources (*e.g.*, cockle, clam, eggshell, and seashell), mammalian bone (*e.g.*, bovine, camel, and horse), marine or aquatic sources (*e.g.*, fish bone and fish scale), plant algae, and also from mineral sources (*e.g.*, limestone) (Fig. 3).^18,34^

Many different methods, such as calcination, hydrothermal, mechanochemical, chemical precipitation, irradiation, and alkaline hydrolysis (Table 1), have been developed and utilized for HA extraction from natural sources in the last decades.^15,18,35–38^ The physical properties of HA highly depend on the source or method from which HA is obtained or synthesized.

**Table 1:** Natural sources of hydroxyapatite

Natural sources of HA		
Mammalian and fish bone	Eggshell and marine and aquatic shellfish	Plants
Calcination over 700 °C	Calcination over 700 °C	Microwave irradiation
Hydrothermal	Hydrothermal	Pyrolysis
Alkaline hydrolysis	Alkaline hydrolysis	Calcination over 700 °C
	Wet chemical precipitation	Hydrothermal
	Mechano-chemical	Alkaline hydrolysis
	Microwave irradiation	Wet chemical precipitation

HA is characterized by low mechanical properties, which limit its application.^6,39–44^ Many papers have reported the possibility of enhancing its mechanical properties through mixing with additives or other materials to form composite materials. For instance, Mobasherpour *et al.* studied the effect of the addition of zirconia and alumina,^45^ while Mukherjee *et al.* studied the effect of nanocarbons on the mechanical properties.^46^ Bose *et al.* used whiskers as a reinforcement agent for its mechanical properties.^47^ Wang and Shaw reported that nanosized HA showed better properties than bulk HA.^48^ HA-based polymer composites have also been synthesized to improve HA's mechanical properties.^49^

**Fig. 4:**
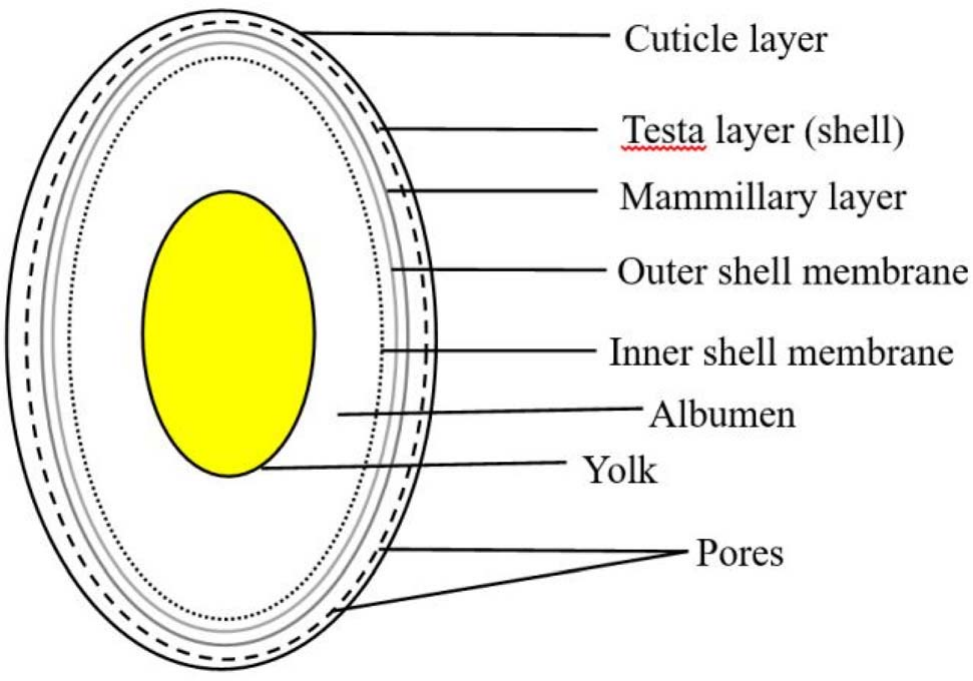
Structure of an eggshell with its various layers.^52^

**Fig. 5:**
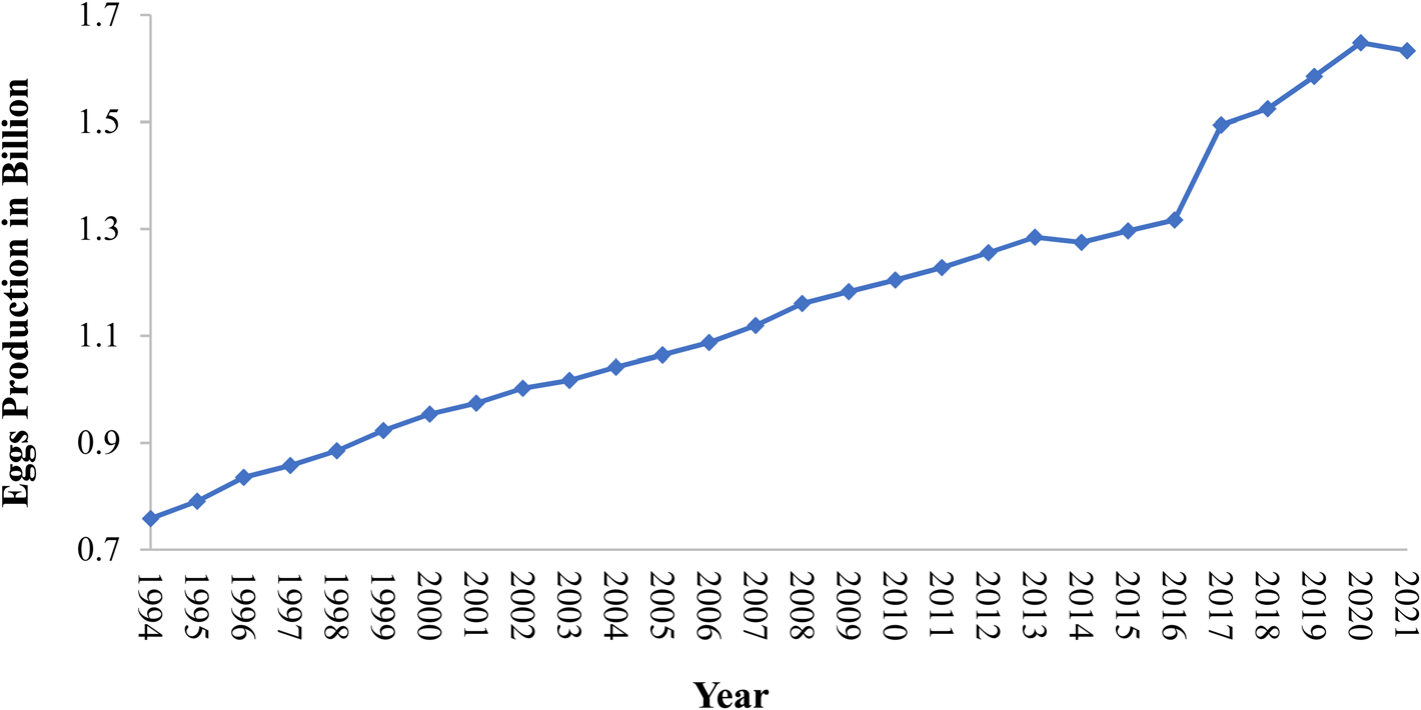
Eggs production growth in recent years.

Eggshell is a common waste material. Eggshell represents around 11% of the total weight of an egg and it is composed of calcium carbonate 94%, calcium phosphate 1%, organic matter 4%, and magnesium carbonate 1%.^50,51^ In general, the eggshell structure (Fig. 4) is composed of three main layers: an outermost layer surrounding the eggshell called the cuticle (about 10 μm), the layer beneath referred to as the testa or palisade layer (about 100 μm), and the innermost layer, termed the mammillary layer. Under the mammillary layer are two shell membranes termed the outer-shell membrane and the inner-shell membrane, where the outer-shell membrane is thicker than the inner-shell membrane, and they together have a thickness of about (100 μm).^52^ Egg-processing industries produce eggshell in large amounts, and to date, high quantities of this solid residue are still disposed of as waste in landfills without any pretreatment, and thus represent a source of organic pollution.^53^ The FAO reported that eggs production increased between 1994 to 2021 from 0.75 billion to 1.63 billion, respectively, with the trend shown below in Fig. 5 highlighting the growth in hen egg production.^54^

According to Waheed *et al.*, the global egg production in 2018 was 78 million metric tons of egg, and as a result of this, approximately 8.58 million metric ton of eggshells were discarded mostly as waste.^55^ In 2020, the FAO published a report revealing that in the past 3 decades years, egg production has grown by more than 150%, and in Asia, egg production has seen even greater progress, increasing by four-fold.^56^ The Environmental Protection Agency (EPA) has ranked eggshell waste as the 15th major food industry pollution problem.^55^ Eggshells as animal by-products are considered a hazardous waste with a low risk as Category 3 in European Union (EU) regulations since they have a risk of spreading or transmitting pathogens such as *Salmonella* to human consumers *via* the food chain.^57,58^ Moreover, the waste can produce emissions of undesired odors arising from ammonia, hydrogen sulfide, and amines as the decomposition products.^52,59^ The EU also considers that washing eggs within production line steps can lead to cuticle thinning and damage to eggshells, increasing the risk of contamination with external bacteria and hindering the shelf life of the eggs.^60^ Most eggshell waste is discarded without further processing by sending it to landfill at a cost of more than $40 a ton depending on the location of the landfill.^44,61–63^

In recent years, the syntheses of HA from natural sources has been extensively studied by researchers.^64^ The abundance of eggshells, the necessity of recycling them to avoid pollution, and the consideration of their elements- and oxides-rich nature have prompted the researchers to study eggshells and investigate the possibility of employing eggshells in important areas as a natural resource. Hydroxyapatite is one of the important products that can be extracted from eggshells and other natural wastes. Commercially available HA is expensive due to the use of high purity of reagents,^65^ so efforts have been directed toward finding an eco-friendly synthesis route for HA from natural sources.^66^ The advantage of HA extracted from natural sources is that it may preserve some of the inherit properties of the raw materials, such as the pore structure and carbonated content.^65^ Interesting experiments (*in vivo*) were conducted by Lee *et al.* to compare synthetic HA and eggshell HA,^67^ and they reported that high-crystalline HA showed higher osteogenic cell proliferation than low-crystalline HA.^68,69^ This result was supported by the XRD patterns, which revealed that eggshell HA was more highly crystalline than synthetic HA.

Wet chemical precipitation is one of common HA synthesis methods, and is characterized by low cost, low operating temperature, and enabling control of the morphology and the mean size of the powder, as well as not requiring an organic solvent.^35,70–72^ From the above-mentioned, this review will highlight the wet precipitation methods to synthesize HA from eggshells. Thus, this review aims to guide researchers and producers to select the optimum conditions to synthesize HA with the desired properties for biomedical applications.

## 2. Wet chemical precipitation method

**Fig. 6:**
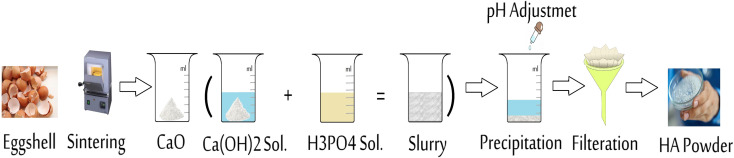
Synthesis of hydroxyapatite from eggshell by wet chemical precipitation.

Hydroxyapatite can be easily synthesized by using a variety of methods, including mechanochemical, chemical precipitation, hydrolysis, sol–gel, and thermal calcination methods. Among them, the wet chemical precipitation method is quite favorable due to its ease in experimental operations, cost-effectiveness, low working temperatures, and high purity of products.^35,73,74^Fig. 6 shows the synthesis of HA from eggshell by chemical precipitation. The wet chemical precipitation method grants researchers better control of the morphology, crystallinity, composition, and synthesis conditions for investigation and studying HA.^75^ Further, the by-products of the precipitation reaction are harmless, and the final product has high purity.^76^ Besides having these advantages, wet methods also have some disadvantages, one of which is that the product HA exhibits low crystallinity owing to the low processing temperatures.^77^

**Fig. 7:**
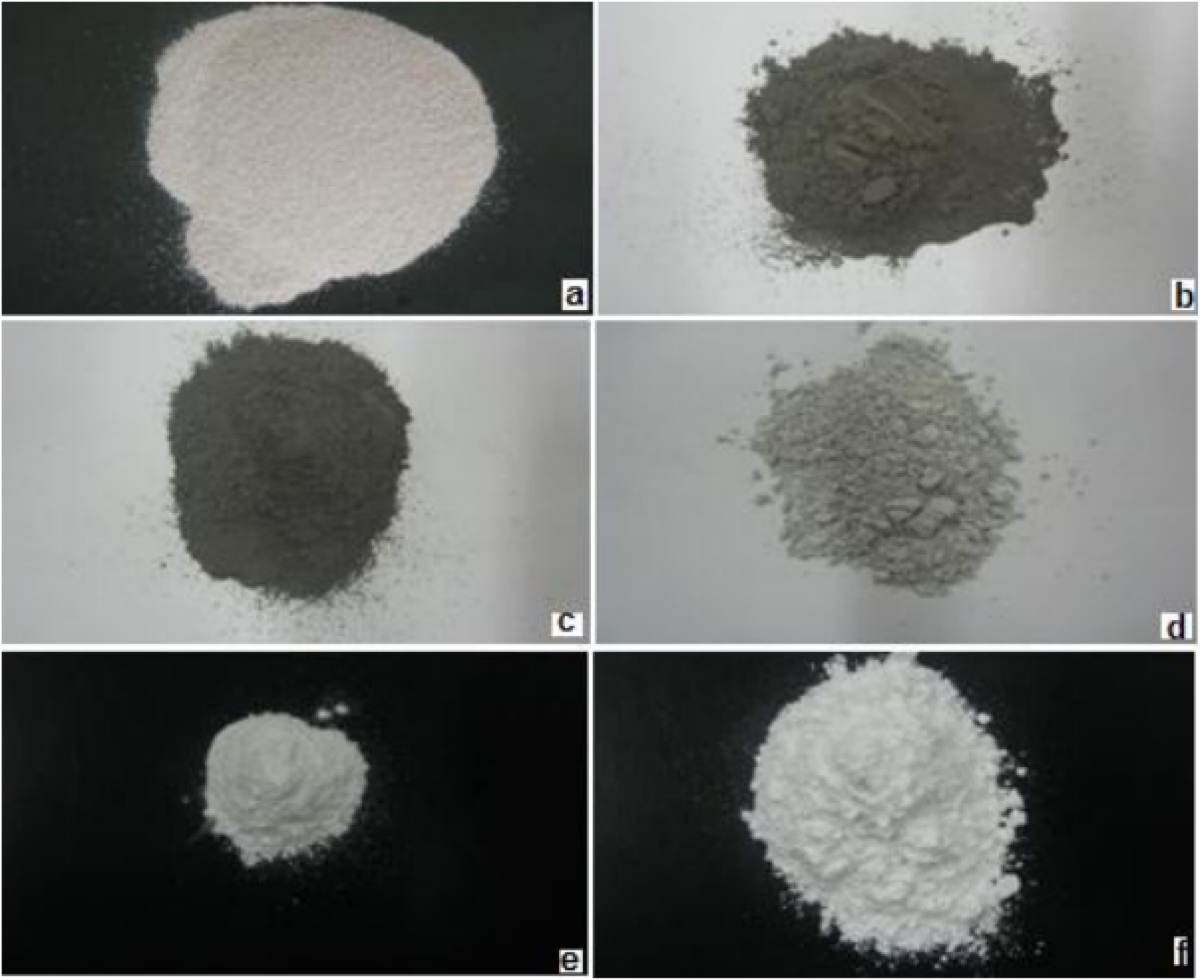
Eggshell powder before being calcined (a) and eggshell powder after being calcined at various temperatures: (b) 600 °C, (c) 700 °C, (d) 800 °C, (e) 900 °C, and (f) 1000 °C.^78^

Some studies used eggshell without removing the organic shell (organic membrane) while others removed it by chemical methods, such as washing the eggshell with diluted HCL. The synthesis of HA from eggshell by a wet chemical precipitation process generally includes deproteinization of the eggshell, precipitation, aging, filtration, drying, and a final heat treatment. The calcination process done at a temperature of up to 700 °C is the most common technique for the deproteinization step. During this first step, CaCO_3_ in the eggshell is reduced into CaO, and CO_2_ is released. The transformation of CaCO_3_ into CaO is accompanied with a color change depending on the variation in the calcination temperature, and Fig. 7 illustrates this relationship from an interesting study. Next, the CaO is dissolved in water to obtain Ca(OH)_2_. In the second step, phosphoric acid is mostly used as a phosphor source to dissolve Ca(OH)_2_ and synthesize HA by precipitation. Some researchers performed experiments at alkaline pH by the addition of drops of NaOH, NH_4_OH, or NH_3_. Other phosphor sources, such as Na_2_HPO_4_ and (NH_4_)H_2_(PO_4_), can also be used to keep the pH at alkaline conditions.

The development of eggshell-based HA synthesized by a wet chemical precipitation method is subject to different routes that typically contain several experiments and process parameters that can affect the properties of the final product, such as the morphology, composition, crystallinity, and particle size. In this section of this review, we present a summary of the studies done in this field starting from 2000. Table 2 presents the summary of the studies using wet precipitation methods to synthesize HA.

**Table 2:** Summary of studies using a wet precipitation method for the synthesis of hydroxyapatite from eggshell

No	Source of PO_4_	pH	Ca/P ratio	Particle size	Morphology	Calcination or irradiation	Ref.
1	H_3_PO_4_	8.5	1.67	10–69 nm	—	400 °C, 700 °C, and 900 °C	79
2	Na_3_PO_4_·12H_2_O and (NH_4_)_2_HPO_4_	—	—	10–100 nm	Circular	—	80
3	(NH_4_)_2_HPO_4_		1.67	18–21 nm	Spherulite	900 °C	65
4	Na_2_HPO_4_·12H_2_O	10	1.69 and 1.5	30–60 nm	Spherical, hexagonal, and cylindrical	900 °C	81
5	(NH_4_)_2_HPO_4_	9	1.64	51.3–82.3 nm	—	1000 °C and 1200 °C	74
6	(NH4)_3_PO_4_	8.6, 9.6, and 10.6	—	—	—	200–1200 °C	82
7	(NH_4_)_2_HPO_4_	9–11	1.425	50–100 nm	Needle-like	—	83
8	(NH_4_)_2_HPO_4_	9	1.67	78.31 nm	Granular	1000 °C	84
9	H_3_PO_4_	7–9	1.71–2.16	30 nm	Globules	400–900 °C	85
10	H_3_PO_4_	9.8–10.4	—	8.9–53.7 nm	Highly interconnected and thick porous particles	200–1000 °C	86
11	H_3_PO_4_	Greater than 10.5	1.68	31.5 nm	—	900 °C	87
12	H_3_PO_4_	10	1.6	0.1–100 μm	Round grains	800 °C	88
13	H_3_PO_4_	—	1.67 ± 0.03	2.5 μm	—	900–1300 °C	89
14	(NH_4_)_2_HPO_4_	10	1.95	33–78 nm	Needle-like	1000 °C	90
15	H_3_PO_4_	8–12	1.67	10–15 nm width and 60–80 nm length	Needle-like and rounded shape (pH dependent)	Irradiation	91
16	Na_2_HPO_4_	11–13	1.37, 1.51, and 1.62	2–3 μm	Flower	Irradiation	92
17	H_3_PO_4_	8.5	1.67	1–27 mm	Irregular particles	700 °C	93
18	(NH_4_)H_2_PO_4_	9	1.49	10–90 nm	Irregular-spherical	250 °C	94
19	Na_2_HPO_4_	10	1.68	38.71 ± 2.1 nm	Oval-shaped	900 °C	95

In 1999, Rivera *et al.* proposed a novel procedure to synthesis porous HA from eggshell.^96^ This procedure included reacting calcined eggshells with phosphate solution in a sealed container at 1050 °C for 3 h. The results confirmed HA formation with traces of other calcium oxides and calcium hydroxide, and the existence of these traces was due to the short reaction time.

In another study, Krishna *et al.* synthesized nanocrystalline hydroxyapatites from eggshells using chemical precipitation, and the followed procedure included reacting sintered eggshells with diammonium hydrogen phosphate solution under microwave irradiation followed by a sintering process.^65^ The results proved that nanocrystalline hydroxyapatite could be successfully synthesized from eggshell waste using microwave irradiation. Compared to synthetic and commercial HAs, the natural nanocrystal HA (length 33–50 nm and width 8–14 nm) seems to have a better morphology, stoichiometry, sinterability, stability at high temperatures, and osteoblast cell adhesion.

Moreover, Ahsan and Ahmed produced Ca-HA from eggshell by a precipitation method.^81^ They studied the use of uncalcined and calcined eggshells in HA synthesis. The micrographs representing the microstructural features of the HA-based uncalcined eggshell exhibited a combination of spherical, hexagonal, and cylindrical shapes having crystal sizes of ∼30–60 nm. Meanwhile the samples of the calcined eggshell did not show clear crystalline particles and a combination of both amorphous and crystalline structures was observed. These results indicate the important role of the calcination step's position in the production lay out.

Goloshchapov *et al.*'s study aimed to find the optimal parameters for the synthesis of nanocrystalline HA by precipitation from eggshells.^85^ Preliminarily, eggshell containing CaCO_3_ was thoroughly washed, and then annealed at 900 °C for 2 h. After that, the obtained CaO was immediately mixed with distilled water at 100 °C and titrated with a solution of orthophosphoric acid to synthesize HA, and then the filtrated product was dried at 400 °C for 1 h. The obtained HA powder granules consisted of nanocrystals with the average size of 30 nm.

In another study, HA powder was synthesized by a wet chemical method, using phosphoric acid (H_3_PO_4_) and eggshells.^97^ The method included cleaning the eggshells, followed by boiling for half an hour, calcination at 900 °C for 1 h to produce CaO, and then again mixing the CaO with water and later with orthophosporic acid. Lastly, the product was filtrated, dried (at 100 °C for 2 h) and sintered again (at 900 °C for 2 h). The synthesized HA powder was crystalline, and the average particle size was about 31.5 nm. The EDX results showed that the Ca/P ratio was around 1.68.

In addition, Neelakandeswari *et al.* in their study performed the synthesis of HA from eggshells using a precipitation method.^83^ First, 1 mmol of calcium chloride dihydrate (CaCl_2_·2H_2_O) was used to adjust the pH (9, 10, 11). Then, diammonium hydrogen phosphate ((NH_4_)_2_HPO_4_) and eggshell membrane were added to the calcium chloride dihydrate (CaCl_2_·2H_2_O) solutions at different pH. The mixture was stirred for 24 h before washing and centrifugation to obtain the precipitate, which was dried to obtain HA powders. According to the results, cylindrical-like shapes with a 5 nm diameter and 50–100 nm length at pH 9 were produced.

Adeogun *et al.* presented a simple method for the synthesis of hydroxyapatite from eggshell material as the calcium source by a wet chemical precipitation reaction with phosphate solution.^90^ The process included collecting and cleaning the eggshells with water to remove dirt and membrane. Deproteinization was next achieved by heating the cleaned eggshells to 450 °C for 2 h. Then, a mechanical grinder was applied to obtain a powder. After that, the obtained powder was dispersed in distilled water and stirred for 30 min with a magnetic stirrer. Thereafter, 100 ml of 0.45 M ammonium dihydrogen phosphate ((NH_4_)H_2_(PO_4_)) was added dropwise and the mixture was stirred for about 30 min. The pH of the solution was adjusted to 10 by the addition of 30% ammonia solution and then left for 24 h for aging under continuous stirring with a magnetic stirrer. The precipitate powder was centrifuged at 4000 rpm, and oven dried at 100 °C overnight to obtain the hydroxyapatite powder. Finally, a calcination process was applied at 1000 °C for 2 h. XRD analysis revealed characteristic peaks corresponding to HAp powder. EDX analysis estimated that the Ca/P molar ratio was 1.95. TEM analysis revealed that the particles had agglomerated needle-like shapes with lengths of 15–60 nm and widths of 4–6 nm.

Goh *et al.* synthesized HA by using a wet chemical method assisted by microwave irradiation at different pH.^91^ The process included calcining eggshells and mixing Ca(OH)_2_ with H_3_PO_4_ and NH_4_OH to adjust the pH. After the reaction was completed, microwave irradiation for 15 min was applied to the mixture in a microwave oven. The obtained white precipitate was washed by distilled water several times, filtered, and finally dried in the microwave oven for 10 min. The formation of HA crystals in the samples synthesized at pH values of 8 to 10 was confirmed by XRD and FTIR analysis. Well-formed needle-like HA particles were obtained at pH 10 approximately 10–15 nm in width and 60–80 nm in length. Further, the formation of more rounded nanoparticles occurred with the increase in the pH.

Pu'ad *et al.* used chemical precipitation followed by a calcination process at different temperatures.^93^ The method included calcining the eggshells at 900 °C followed by finely grinding them. Next, the calcined eggshells were added to distilled water, and then phosphoric acid (H_3_PO_4_) was added to the solution until the pH value was set at 8.5. The solution mixture was then left to age for 24 h at ambient temperature. The solution was then re-stirred for another 30 min, and left for another 24 h to complete the precipitation process. The solution was then filtered and rinsed before drying at 100 °C. The dried precipitate was again calcined for 2 h at various temperatures: 300 °C, 500 °C, 700 °C, 900 °C, and 1100 °C. The results showed that crystalline HA was synthesized from the combination of chemical precipitation and the calcination method utilizing eggshell as the calcium precursor. The calcination temperature of 700 °C showed the best results, whereby crystalline HA with a Ca/P ratio 1.67 was synthesized and the particle size ranged from 1 to 27 mm. Biphasic HA and tri calcium phosphates were observed at calcination temperatures of more than 700 °C.

In conclusion, in this method several parameters, such as the pH, sintering, reaction temperature, irradiation, aging time, and doping elements, can influence the structure and properties of the synthesized hydroxyapatite. Therefore, the reproducibility of HA with the same size and properties depends on a good control and stability over the synthesis. Also, every method has its advantages and limitations.

## 3. Parameters affecting the properties of hydroxyapatite synthesized by the wet chemical precipitation method

Chemical precipitation routes are highly affected by the parameters and conditions that control the reaction. In this section, we discuss the reaction parameters and their effects on the properties of the synthesized HA.

### 3.1. pH

**Fig. 8:**
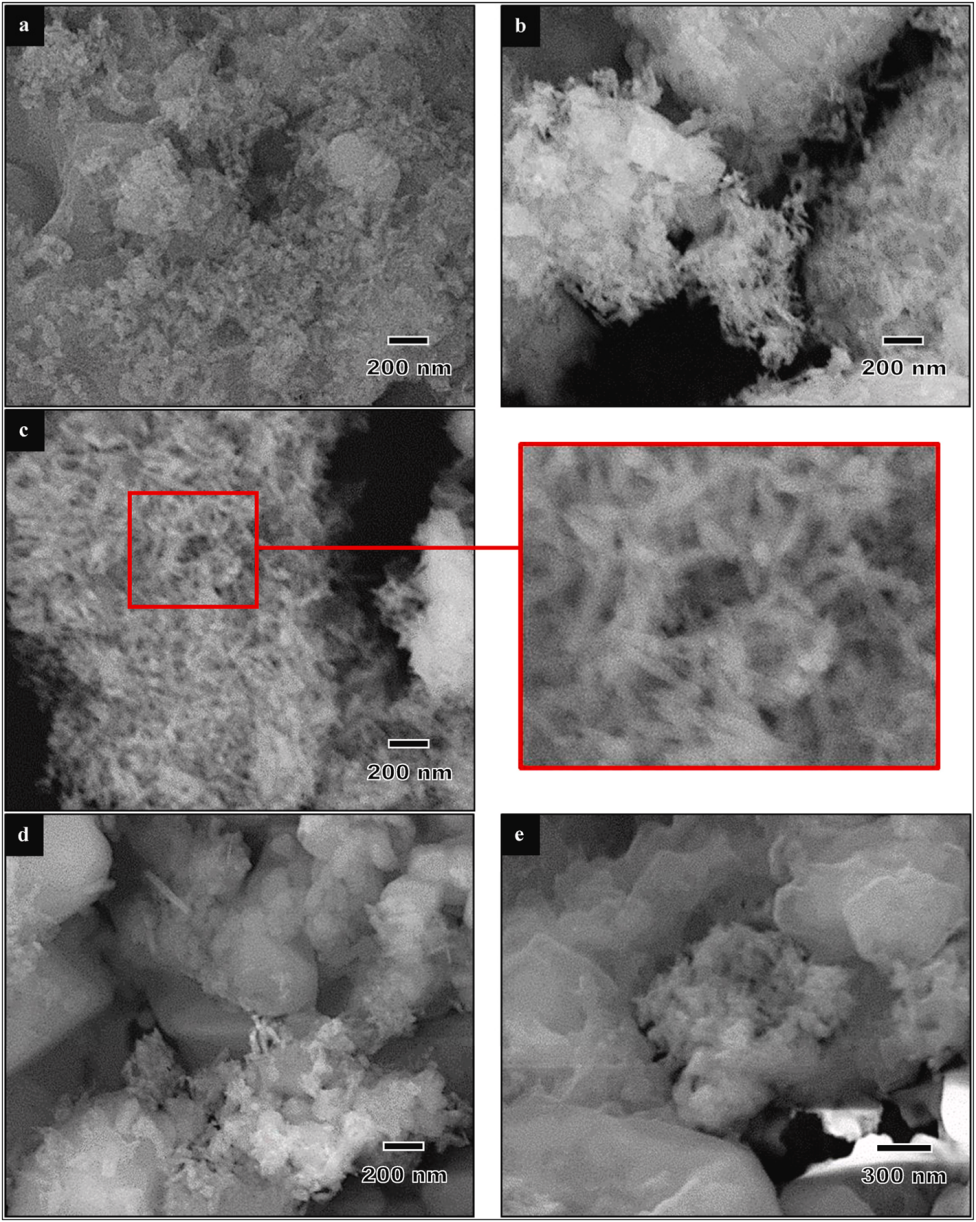
FESEM micrographs of eggshell-derived hydroxyapatite produced at different pH values: (a) 8, (b) 9, (c) 10, (d) 11, and (e) 12.^91^

The synthesis of HA by the chemical precipitation method is affected by the pH of the reaction. Therefore, drops of a basic solution like NaOH, NH_4_OH, or NH_3_ are used to adjust the pH. However, in some studies, pH adjustment was not done using such pH adjustor chemicals.^93^ Goh *et al.* studied the effect of the pH on eggshell-based HA synthesized by chemical precipitation methods.^91^ They reported that well-formed needle-like HA particles were obtained at pH 10 that were approximately 10–15 nm in width and 60–80 nm in length, and while a further increase in the pH (11 and 12) resulted in the formation of more rounded nanoparticles (Fig. 8). This result was consistent with that of Amiruddin and Noor's study, confirming that the synthesis of HA under acidic conditions produced spherical and rod-like shaped particles whereas HA synthesized at pH 9 showed rod-shaped particles.^98^ Previously, Goloshchapov *et al.* discovered that different pH levels of the reaction affect the thermal stability of HA.^85^ They reported that HA synthesized at pH 7–7.5 completely decomposed into whitlockite at the annealing temperature, meanwhile HA synthesized at a higher pH retained the phase of HA simultaneously with a certain amount of whitlockite at 900 °C. Neelakandeswari *et al.* observed that an increase in the pH of the reaction medium from 9 to 11 corresponded with an increase in the crystallite size along the *a*-axis.^83^ These observations shed light on the morphology change with pH. Núñez *et al.* found that the pH influenced the surface electrical properties of the charged porous media.^99^ When HA was suspended in water, the resulting pH ranged between 7.3 and 8.7 with negative zeta potential values. On the other hand, the values shifted to positive when the pH of the HA suspension was adjusted to 5.0. The effect of the pH over the surface charge is an essential parameter regarding biomedical or environmental applications (*e.g.*, heavy metal removal). Pankaew *et al.* studied the synthesis and phase transformation of biphasic materials (HA and β-TCP), and confirmed that β-TCP was enhanced at pH from 8.6–10.6.^82^ Muthu *et al.* reported that a pH above 9 and Ca/P ratio of 1.67 with vigorous stirring led to *in situ* amorphous HA formation, but aging or exposure to microwaves resulted in an improvement in HA crystallinity.^92^ In the same paper, they reported that increasing the pH value led to a relatively increased yield of HA. In general, calcium-deficient hydroxyapatite may be formed upon processing at a pH lower than 9.^73,100^

### 3.2. Sintering

**Fig. 9:**
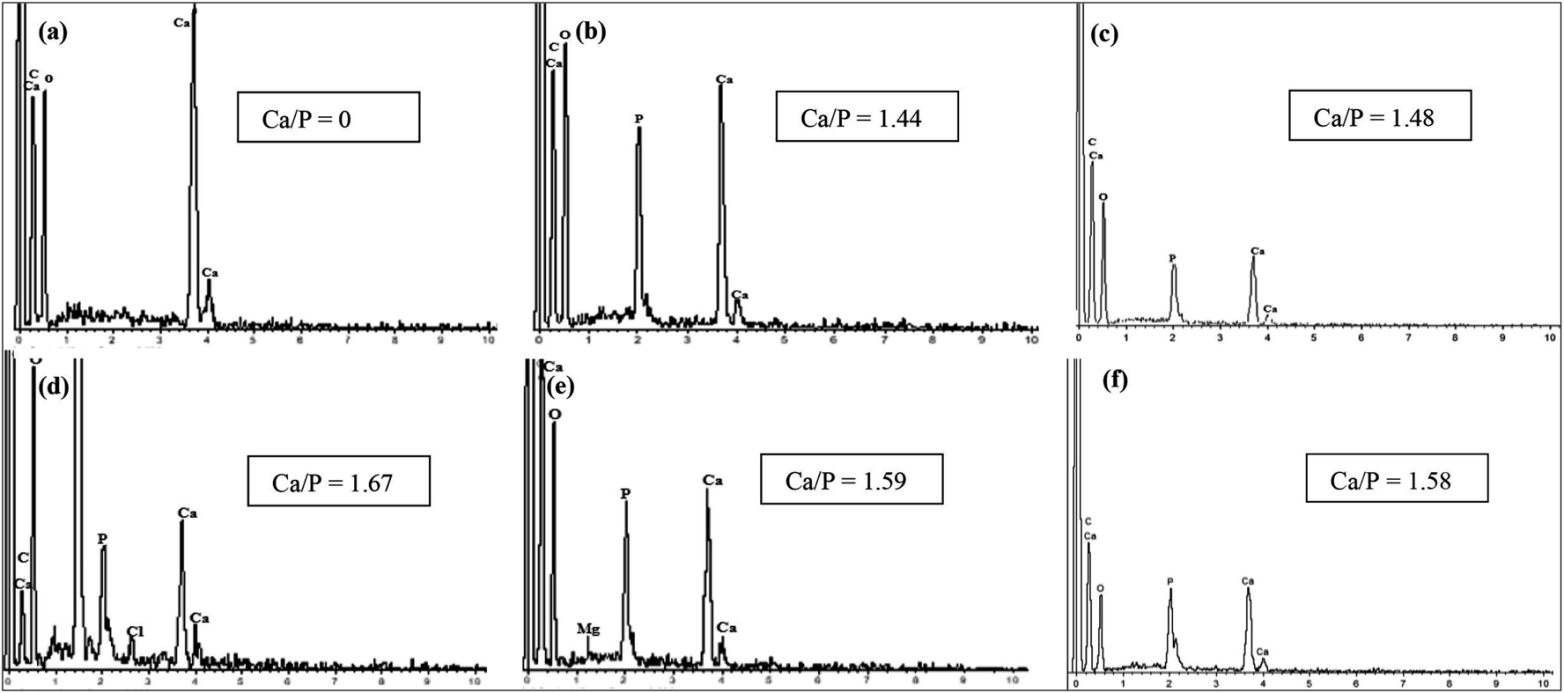
EDS analyses of (a) calcined raw eggshell (900 °C) and all the synthesized samples ((b) 300 °C, (c) 500 °C, (d) 700 °C, (e) 900 °C, and (f) 1100 °C).^93^

The sintering process is done by heating HA at high temperature in an open atmosphere furnace. Sintering has been extensively studied in the synthesis of HA. The aim of using sintering is to improve the physical properties and crystal structure of the product. The thermal stability of eggshell-based HA was observed by Krishna *et al.* and Kamalanathan *et al.*, and they found that HA decomposed at 1250 °C and released CaO.^65,87^ Sintering at a temperature below 1250 °C was accompanied with a sharpening of the XRD peaks, but after 1300 °C, the intensity of the peaks became wider.^87^ In another study, an increase in the XRD peak intensity of HA derived from eggshell occurred as the sintering temperature was increased until its decomposition temperature due to the crystallinity.^93^ The transformation of HA into other phases or the appearance of a biphasic material were observed at sintering temperatures of more than 900 °C,^93^ 1200 °C,^65^ and 1250 °C,^87^ variously. Roudan *et al.* revealed that eggshell-based HA presented thermal stability even at 1300 °C.^89^ Pu'ad *et al.* suggested that 700 °C was the optimum sintering temperature, and higher temperature led to biphasic HA and β-tricalcium phosphate.^93^ The ratio of Ca/P changed with the sintering temperature, and this was attributed to the formation of a biphasic material (Fig. 9). There is almost unanimity among most researchers that sintering improves the hardness, density, and toughness.^65,87,89^

Also, the Vickers' hardness increases with the sintering temperature increasing until reaching decomposition temperature, and after this point, the hardness decreases. Kamalanathan *et al.* reported that the Vickers' hardness is governed by the relative density (which is governed by the sintering temperature) until the grain growth reaches a critical point.^87^ After this point, hardness is dictated by grain growth. The morphology of HA is influenced by the sintering temperature.^85^ The average sizes of HA particle conglomerates is increased after the annealing process. The morphology of sintered HA reported in the literature is a needle-like shape.^87^

### 3.3. Reaction temperature

**Fig. 10:**
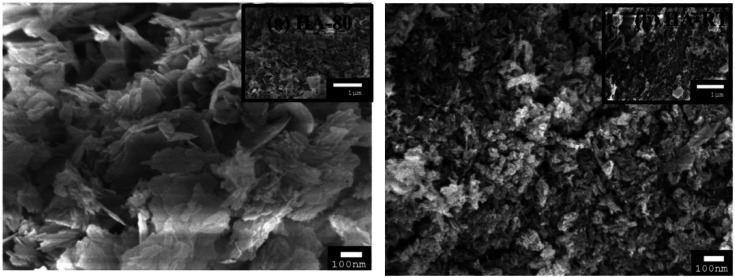
SEM images of (a) HA synthesized at 80 °C and (b) HA synthesized at RT. HA-80 has a flake-like morphology and HA-RT has a somewhat nanorod-shaped morphology, and the particles of HA-80 are larger than the HA-RT particles.^101^

The reaction temperature is such a critical parameter that it should be considered in the synthesis of HA. In the wet chemical precipitation method, Saha *et al.* pointed out that there is a relationship between the reaction temperature and properties, including the morphology, particle size, and Ca/P ratio (Fig. 10).^101^ They also reported that a high reaction temperature yields flake-shaped products with particle sizes of 55–68 nm, whereas rod-shaped ones with particles sizes between 23–32 nm were obtained at room temperature. It is believed that a high reaction temperature stimulates crystallization growth. Rivera *et al.* synthesized eggshell-based HA at reaction temperatures reaching 1050 °C for 3 h, and for the reaction, they used a container designed to reproduce a moist atmosphere.^96^ Finally, they produced a solid material with a porous texture, white color, high mechanical strength, and pores with irregular diameters. Yelten-Yilmaz and Yilmaz believed that the solubility of Ca(OH)_2_ in phosphoric acid decreases with increasing the reaction temperature, leading to an impedance in the precipitation continuity of HA formation.^71^ Yelten and Yilmaz agreed with Saha *et al.* in terms of the crystal size, thus stating that a high reaction temperature stimulated crystal growth, leading to crystal sizes larger than the crystals synthesized at room temperature.^72,101^ Large crystal sizes are not preferable for biomedical applications because crystal growth corresponds with the pore size decreasing, which can negatively affect cell adhesion and proliferation in implants with tissues. Bardhan *et al.* succeed in producing HA at 55 °C by reacting Ca(NO_3_)_2_ (derived from uncalcined eggshell) and (NH_4_)_2_H(PO_4_), but they observed porosity between (10–12%) in the product.^74^ In the same year, similar chemicals, despite using calcined eggshell, were used to prepare HA at a reaction temperature of 100 °C.^82^ It should be noted that HA prepared *via* low temperature wet chemical processes is known to accommodate various ionic species, *e.g.*, H_3_O^+^, H(PO_4_)_2_^−^, *etc.*, and they may decompose in a variety of ways, leading to structural changes.

### 3.4. Irradiation

**Fig. 11:**
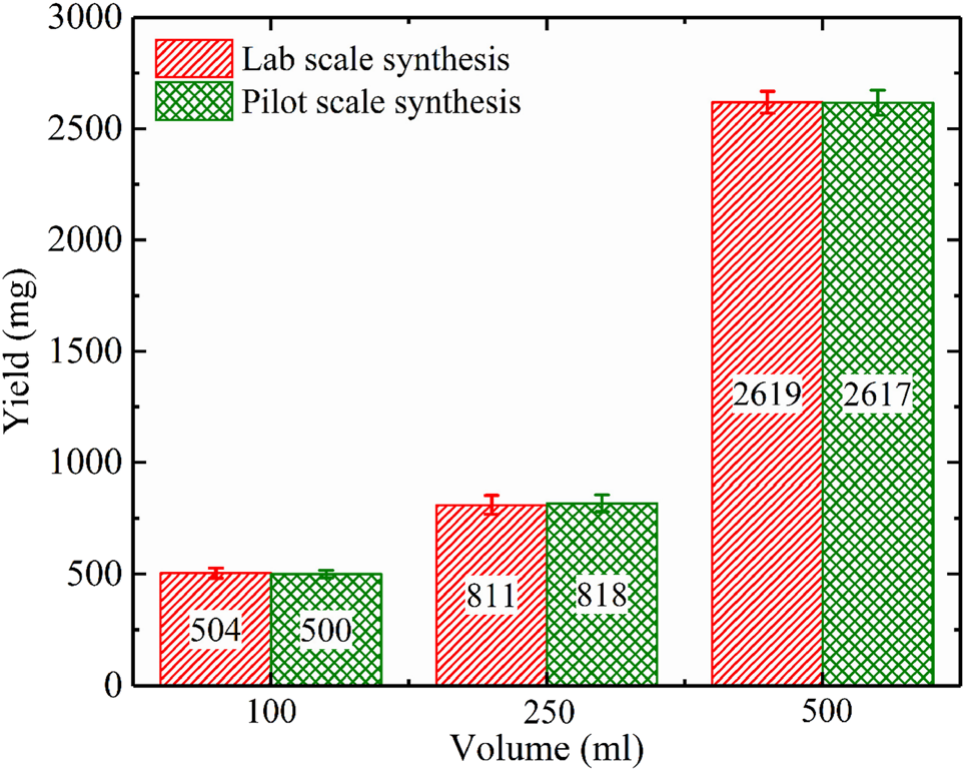
Comparison of the yield of the product against volume for lab-scale and pilot-scale microwave syntheses.^92^

Another technique to increase the reaction rate rather than the temperature is to use microwave irradiation.^65^ The wet chemical precipitation method assisted by microwave irradiation has been performed by some researchers recently.^65,91^ In Kumar *et al.*‘s work, eggshell biowaste was utilized to produce a flower-like hydroxyapatite nanostructure by means of a microwave irradiation method with the help of ethylenediaminetetraacetic acid (EDTA) as a chelating agent.^102^ The process did not include eggshell calcination and chemically treating in sodium hypochlorite (NaClO) to remove the organic parts. Then, the eggshell powder was mixed with EDTA solution to form a Ca-EDTA complex, and a certain amount of Na_2_HPO_4_ solution was added to the Ca-EDTA complex and stirred for 30 min. The pH of the reaction mixture was adjusted to 13 by the addition of NaOH solution. Then, the mixture was treated in an irradiated microwave for 10 min. The role of EDTA was to control the growth of HA crystallites (using the Ca-EDTA complex stability) and the adsorbed OH– ions on the facets of the crystallites. SEM observations exhibited the formation of flower-like nanostructured HA 100–200 nm in width and 0.5–1 μm in length. The XRD patterns confirmed the formation of pure HA. Goh *et al.* studied the effects of applying irradiation of 700 W for 15 min at different pH during wet chemical precipitation.^91^ They observed a three-dimensional cluster was formed, which acted as nuclei for HA crystals during the initial stage that grew into crystallites with the microwave irradiation. Also, they proved that HA powder could be obtained immediately in aqueous solution under microwave irradiation, and there was no phase transformation to amorphous calcium phosphate during the microwave irradiation. Applying microwave radiation allows time saving.^91^ Muthu *et al.* studied irradiation for lab-scale (900 W) and pilot-scale (2.2 kW) production, and the results from the pilot-scale reactor revealed that although the microwave power from the pilot-scale reactor was considerably higher than that of the lab-scale reactor, there was no significant difference in the yield of the product, except that the microwave irradiation time was reduced considerably, almost in proportion to the power of the reactor (Fig. 11).^92^

### 3.5. Aging time

In the synthesis of HA, the aging time is another important factor as it improves the thermal stability of HA and also facilitates crystal structure perfection.^103^ The aging process helps in enhancing the kinetics of HA crystals formation, thereby helping in crystal growth.^104^

**Fig. 12:**
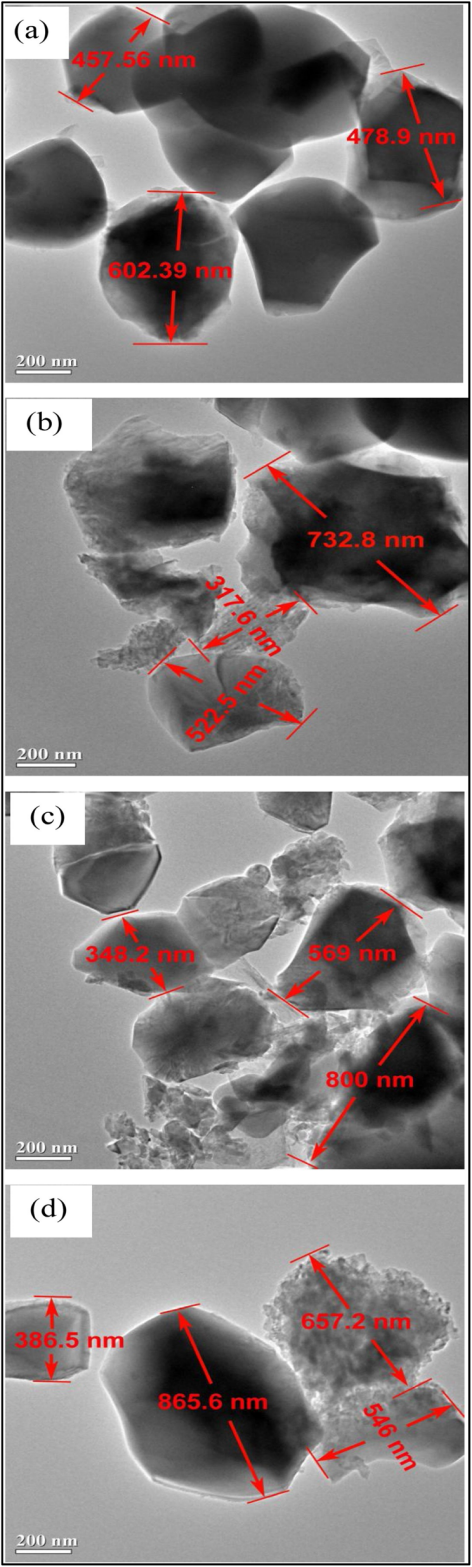
TEM micrograph of the synthesized HA with (a) 12 h, (b) 24 h, (c) 36 h, and (d) 48 h aging time, respectively.^95^

It was reported that highly crystalline HA improves the osteoconduction and osteointegration required for biomedical applications.^105^ Researchers discovered a relationship between the surface area and aging time.^106^ Lala *et al.* studied the effect of aging time on HA, and they reported that there is a relationship between the relative density and shrinkage *versus* the aging time.^95^ Also, the relative density was found to increase with the increase in the aging time. They confirmed that the increase in aging time led to an increase in crystallite size due to the cross-linked structure of the molecules and the enhancement of the kinetics of formation of HA crystals, thereby increasing the crystal sizes of the HA particles (Fig. 12). One of the important parameters to consider for biomedical applications is the porosity of HA. It should be mentioned that increasing the HA crystal sizes by aging also corresponds with decreasing the pore size, and this is not favorable for scaffold and implant materials, as closed pores do not allow cell adhesion and proliferation. Crystal growth caused by aging is displayed in the XRD pattern peaks as a shifting in the crystallography. Agbeboh *et al.* concluded that the phosphorus resource affected the aging time required for HA formation.^107^ For instance, orthophosphoric acid required a longer aging time of about 7 days, meanwhile only 24 hours aging time was enough for the nitric acid and diammonium hydrogen phosphate-based wet precipitation method. Odusote *et al.* studied the effect of aging time over the Ca/P ratio, and they found that at 24 hours aging time, the Ca/P ratio was 1.67, while at lower aging times (0 and 6 h), unreacted CaO was observed.^108^

### 3.6. Doping elements

**Fig. 13:**
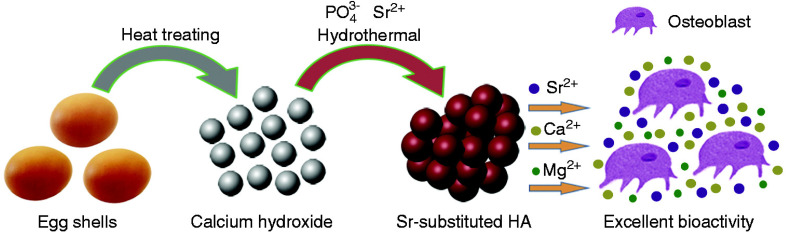
Schematic of the hydroxyapatite synthesis process and the effect of cations on osteoblast growth.^111^

Hydroxyapatite (HA) in natural bone is non-stoichiometric due to the presence of impurities, such as magnesium, potassium, and sodium.^109^ Therefore, to mimic human bone, synthesizing HA with various substituted elements is preferable. Significant efforts have been made to modify synthetic HA to enhance its solubility, bioactivity, and osteoinductivity. Such modification includes the incorporation of various ionic elements, such as iron (Fe^2+^), calcium (Ca^2+^), magnesium (Mg^2+^), strontium (Sr^2+^), zinc (Zn^2+^), and manganese (Mg^2+^).^110^ An interesting study confirmed that when Sr was incorporated with HA based on eggshells, it led to a slight decrease in crystallinity and specific surface area while further enhancing the bioactivity.^111^ The same study reported that HA produced from eggshells and Sr^2+^ as the second element could be a promising biocompatible material for future clinical applications (Fig. 13). Another group studied the incorporation of Mg and Sr in HA, reporting that only a limited amount of Mg could successfully substitute for Ca in HA, resulting in reduced crystallinity, thermal stability, and lattice parameters of HA, while Sr could fully substitute for Ca.^109^ Furthermore, the addition of Sr increased the lattice parameters of HA. There is a consensus that Sr improves the biological properties of HA.^109,111,112^ The incorporation of Zn into HA based on eggshells was studied, and the results confirmed that the powders were highly crystalline after calcination at 700 °C despite the Zn substitution. The Zn-doped powders indicated the presence of a dual-phase structure comprising HA as the major phase and β-TCP as the minor phase.^110^ The effect of Mg concentration on the mechanical strength of HA based on eggshells was also studied, and the authors found that Mg enhanced the hardness of HA doped with Mg, attributed to changes in its lattice parameters and morphology.^41^

## 4. Outlook and challenges

Eggshell-based hydroxyapatite (HA) synthesis by wet chemical precipitation is a highly sensitive method. Thus, the parameters and conditions for conducting wet chemical precipitation should be strictly controlled and monitored throughout all the synthesis steps. Regarding eggshell heat treatment, the optimal deproteinization temperature reported is 900 °C; lower temperatures are not recommended for the complete transformation from CaCO_3_ to CaO. Although the Ca/P ratio is stoichiometrically predetermined, achieving HA with a Ca/P ratio of 1.67 depends on other reaction parameters. Studies suggest that a pH range of 9–10 is optimal for obtaining nanocrystalline HA, with increasing pH leading to larger crystal sizes, while there is the formation of calcium-deficient hydroxyapatite when processing at pH levels lower than 9. The sintering process applied to HA enhances its crystallinity at temperatures between 700 °C and 900 °C, but biphasic peaks are observed beyond this. Regarding the reaction temperature, it is agreed that high temperatures accelerate crystal growth toward larger diameters beyond the nanoscale, although the solubility of Ca(OH)_2_ in phosphoric acid decreases with increasing the reaction temperature, hindering the reaction progression. Utilizing microwave irradiation as a catalyst is feasible for HA synthesis, reducing the time, and there is no significant difference between irradiation at 700 W and 900 W. An aging time of 24 h is considered the optimal aging period; longer aging times lead to lower porosity and increased relative density, while unreacted CaO is observed at lower aging times (0 and 6 h).

Although a lot of satisfactory progress has been made in this field in the last decades, comprehensive information on eggshell-based HA obtained *via* the wet chemical precipitation method is still scarce. First of all, based on previous research, the decomposition behavior of eggshells before and after calcination, particularly in the complete decomposition of CaCO_3_ to CaO and prior to synthesis, must be analyzed and confirmed since undecomposed eggshell may influence phase changes. Another challenge is that the calcination of eggshell includes a release of CO_2_, which causes pollution and consumes a lot of energy, so green alternative methods for deriving Ca(OH)_2_ from eggshell should be found and highlighted in future research. Besides, the content of CaCO_3_ in eggshells is dependent on the vital activities of the hens. Thus, eggshells collected from hatching have a low calcium content compared to food eggs. To this end, it is highly essential to overcome these obstacles through some effective strategies.

The phosphorous source is extremely important for the microstructure of the HA. In the literature, all PO_4_ anions used in wet chemical precipitation were derived from chemicals like H_3_PO_4_ and/or phosphoric salts like Na_2_HPO_4_. In the future, it is recommended to use phytic acid as a green source of phosphor because phytic acid has six-fold dihydrogen phosphate ester of inositol. Moreover, using Na_2_HPO_4_ as a source of phosphor results in Na cations that may be used in the alkali reaction for producing composites of HA/geopolymers.

Last but not least, the synthesis of hydroxyapatite from eggshell is not only the main concern: the mechanical, biological, and physical properties are also important for employing hydroxyapatite to meet the demands of the intended practical applications. Therefore, the final properties should be targeted to suit these applications. In consideration of this point, wet chemical precipitation enables manufacturers to insert catalysts, additives, and reinforcement agents to enhance the HA yield and physiochemical properties. Further research on the incorporation of elements into the structure of hydroxyapatite, along with exploring the use of additives containing certain elements to enhance the mechanical properties is also needed. By integrating additives such as kaolinite, slag, and fly ash into hydroxyapatite based on eggshells through a wet chemical precipitation method, remarkable mechanical enhancements can be potentially achieved. These materials, often regarded as waste, offer promising opportunities for improving the mechanical specifications of HA when used in specific proportions and conditions.

## 5. Conclusion

Day by day, the demand and usage of hydroxyapatite in biomedical applications, such as dental implants, dental fillings, and bone restoration, are increasing. This makes it an ever-more valuable material. Furthermore, there is an urgent need to transform it into a cheap and environmentally friendly biomaterial that can replace expensive biomedical materials that are currently used in biomedical applications.

In this review, eggshell-based hydroxyapatite has demonstrated great potential as an alternative HA in terms of its material properties. Among the methods used to synthesize HA, the wet chemical precipitation method is quite favorable due to its ease in experimental operations, cost-effectiveness, low working temperatures, and high purity of products. The different routes involved in the wet chemical precipitation method have effects on the physical and chemical properties of the HA obtained using eggshells. Both phosphate precursors and the process parameters during the synthesis of HA influence the Ca/P ratio, particle size, morphology, and crystallinity.

## Conflicts of interest

There are no conflicts of interest to declare.
